# Adsorption of As(III), Pb(II), and Zn(II) from Wastewater by Sodium Alginate Modified Materials

**DOI:** 10.1155/2021/7527848

**Published:** 2021-09-23

**Authors:** Hongchuan Li, Rui Jin, Hongxiang Hu, Yusef Kianpoor Kalkhajeh, Yingying Zhao, Yue Gao, Borui Zhang

**Affiliations:** School of Resources and Environment, Anhui Agricultural University, Hefei 230036, China

## Abstract

Sodium alginate (SA), polyvinyl oxide (PEO), and ceramic nanomaterials were used to prepare alginate composite gel. The present study examined the removal rate and adsorption capacity of alginate composite gel for removal of wastewater As(III), Pb(II), and Zn(II). Batch experiments were conducted to study the influence of experimental parameters such as pH and temperature, as well as the mechanism of As(III), Pb(II), and Zn(II) adsorption with the new adsorbent. The results showed the high efficiency of sodium alginate composite gel for removal of wastewater As(III), Pb(II), and Zn(II). Under the condition of the best liquid-solid ratio and the contact time, the removal rates of As(III), Pb(II), and Zn(II) were 67.42%, 95.31%, and 93.96%, respectively. The pseudo-second-order kinetic equation was superior to fit the adsorption kinetics process. The isothermal adsorption models of As(III) and Pb(II) fitted well with the Freundlich model, and Zn(II) fitted well with the Langmuir model. The results of SEM, EDS, XPS, and FTIR analyses revealed that the adsorption process occurred mainly via chemisorption. The results of the present study suggest that new adsorbents can be effectively utilized for As(III), Pb(II), and Zn(II) removal from water.

## 1. Introduction

With the rapid development of industry in recent decades, heavy metals have been discharging into the water bodies in high quantities threatening water quality. Arsenic is the world's number one teratogenic carcinogenic toxin [1]. At drinking water arsenic concentration of 50 mg L^−1^, people are at risk to various cancers such as liver cancer, bladder cancer, and skin cancer [2]. Lead is also one of the most toxic heavy metals which greatly reduce the activity of several proteins, amino acids, enzymes, and other vital substances in human body. In particular, children are vulnerable to lead poisoning as they may suffer from loss of appetite, stomachache, constipation, diarrhea, insomnia, and learning disabilities [3]. Although zinc is a necessary trace element to promote human growth and maintain health, in excessive amounts it might irritate the digestive tract and affect nutrient absorption. Simultaneously, high amount of zinc inhibits the role of copper and iron in human body, resulting in metabolic disorders. Therefore, it is of major importance to remove these toxic elements from wastewater, reducing their potential health risks to individuals. At present, coagulation and precipitation, ion exchange, biological treatment, and adsorption are the most common methods to remove heavy metals from water resources [4]. Compared with coagulation and precipitation, ion exchange, and biological methods, adsorption method is a highly capable and time-efficient technique with simple operation [5]. Different from traditional non-model adsorption materials, natural polymer adsorption materials not only have better adsorption effects, but also have less impact on the environment [6–8]. Sodium alginate gel is a superior adsorbent for wastewater heavy metals that combines sodium alginate [9, 10] with other materials. Zeng et al. [11] used alginate and chitosan to preparing a granular adsorbent for sludge containing iron/manganese in an arsenic adsorption study. Lee et al. [12] granulated CMD sludge (CMDS) into polyurethane to remove arsenic/heavy metals. Lin et al. [13] prepared sodium alginate-polyvinyl oxide gel spheres with high removal rates of Pb(II), Cu(II), and Cd(II). Hu et al. [14] used the cross-linking method to prepare arylated cellulose nanocrystalline sodium alginate hydrogel microspheres with an adsorption rate of 76% for Pb(II). Karthik et al. [15] studied the ability of Ca^2+^ cross-linked sodium alginate beads to remove Cr(VI) from aqueous solution with a maximum adsorption capacity of 24.2 mg g^−1^. Pan et al. [16] and Wen et al. [17] modified sodium alginate to significantly improve its adsorption capacity. When sodium alginate, polyvinyl alcohol, and porous ceramic nanomaterials are used for wastewater adsorption alone, the effect is unstable and the removal rate is not high. Thus, further investigation to prepare and apply sodium alginate gel adsorbents with high adsorption capacity and environmental friendliness is of great significance for practical environmental remediation. In this paper, sodium alginate, polyvinyl oxide, and porous ceramic nanomaterials were combined to prepare a composite material with strong adsorption effect for removal of wastewater As(III), Pb(II), and Zn(II). The efficacy of sodium alginate gel was explored in relation to the properties of raw materials, solution pH, temperature, reaction time, adsorbent dosage, solution concentration, and the adsorption mechanism.

## 2. Materials and Methods

### 2.1. Experimental Materials and Instruments

Sodium alginate (SA) and polyvinyl oxide (PEO) were purchased from Hefei Bomei Biological Technology Co., Ltd. Multi-void ceramic nanomaterials were purchased from Wuhu Gefeng Co., Ltd. As(III) stock solution was prepared from sodium arsenite (NaAsO_2_, guaranteeing 99.9% reagent grade, Hubei Hengjingrui Chemical Co., Ltd., Wuhan, China). Lead nitrate (Pb(NO_3_)_2_, Guaranteed Reagent Grade 99%, Aladdin Co., Shanghai, China) was used to prepare Pb(II) stock solution; Zn(II) stock solution was prepared from zinc nitrate hexahydrate (N_2_O_6_Zn·6H_2_O, Reagent Grade 99%, Aladdin Corporation, Shanghai, China). All other reagents (HCl, Ca(NO_3_)_2_·4H_2_O, HNO_3_, NH_4_OH, acetone, etc.) were used without further purification.

Instruments were as follows: acidity meter (Starter 3100); coolable thermostatic vibrating screen (IS-RDD3) for temperature regulation and control; low-speed centrifuge (SC-3610); inductively coupled plasma emission spectrometer (ICAP 7000 Series, Thermo Fisher, USA) used to determine the concentrations of As(III), Pb(II), and Zn(II) after adsorption; S-4800 scanning electron microscope (SEM, Hitachi, Japan); in situ x-ray photoelectron spectroscopy (NEXSA, Thermo Fisher, USA); Fourier Transform Infrared Spectrometer (Thermo Fisher, USA).

### 2.2. Experimental Methods

#### 2.2.1. Synthesis of Composite Materials

5 g sodium alginate, 2.5 g polyvinyl oxide, and 10 g porous ceramic nanomaterials were mixed evenly, dissolved in 200 ml ultrapure water, and stirred uniformly. At room temperature (25°C), the mixed solution was dropped into 1L Ca(NO_3_)_2_·3H_2_O solution (0.3 mol L^−1^) to prepare sodium alginate composite hydrogel blocks, which were placed overnight to stabilize and continuously entered into 200 ml acetone aqueous solution (8%, v/v) for 24 h to complete solvent exchange.

#### 2.2.2. Stock Solutions

Standard solution: sodium arsenite, lead nitrate, and zinc nitrate hexahydrate were mixed with pure water to prepare stock solutions of As(III), Pb(II), and Zn(II) with a concentration of 200 mg L^−1^. pH was adjusted to 1∼10 using HCl and NH_4_OH.

#### 2.2.3. Characterization of Materials

The microstructure of the materials before and after adsorption was observed by SEM scanning electron microscopy; the changes of elements before and after adsorption were detected by EDS electron microscopy; the molecular structure and valence state of the materials before and after adsorption were analyzed and compared by XPS; and the surface functional groups were measured by FTIR.

#### 2.2.4. Batch Experiment

In this experiment, sodium alginate gel was added at dosages of 1∼20 g; oscillation time of the kinetics experiment was set at 30∼300 min; initial concentration isotherm adsorption experiments ranged from 50 to 1000 mg L^−1^; pH was adjusted to 1∼11; and the temperature was adjusted to 15∼40°C. The rotating speed was 150 r/min and the vibration was 180 min. The obtained solutions were centrifuged at 3000 R for 10 min. After filtration, the concentrations of residual As(III), Pb(II), and Zn(II) in wastewater were determined by inductively coupled plasma spectrometer. According to the concentration of As(III), Pb(II), and Zn(II) in the residual wastewater after equilibrium adsorption, the equilibrium adsorption amount was calculated and the effects of various factors on the adsorption of As(III), Pb(II), and Zn(II) by sodium alginate composite gel were analyzed.

### 2.3. Calculation and Model

The formulae used to calculate adsorption capacity (1) and removal rate (2) are as follows:

(1) $$\begin{array}{c} q=\frac{\left(\rho -\rho_{i}\right)V}{W}, \end{array}$$

(2) $$\begin{array}{c} U=\frac{\left(\rho -\rho_{i}\right)100\%}{\rho }, \end{array}$$

where *q* is the adsorption capacity of sodium alginate mixed gel for wastewater As(III), Pb(II), and Zn(II) (mg g^−1^); *U* is the removal rate (%); *ρ* and *ρ*_*i*_ are the initial concentrations of As(III), Pb(II), and Zn(II) in wastewater and the concentration at equilibrium of adsorption, respectively (mg L^−1^); *V* is the volume of solution (L); and *W* is that the weight of the adsorbent (g).

The pseudo-first-order kinetics model and pseudo-second-order kinetics model were used for fitting calculation of the adsorption kinetics model [18].

The pseudo-first-order kinetics model is as follows:

(3) $$\begin{array}{c} \text{Langmuir model}=q_{t}=q_{e}\left[1-\exp\left(-k_{l}t\right)\right]. \end{array}$$

The pseudo-second-order kinetics model is as follows:

(4) $$\begin{array}{c} q_{t}=\frac{q_{e}^{2}k_{2}t}{1+q_{e}k_{2}t}, \end{array}$$

where *q*_*t*_ and *q*_*e*_, *k* are adsorption capacity at time *t* and equilibrium time, kinetic constant, respectively. In the fitting process, the model is judged by determination coefficient (*R*^2^) and Chi-square value (*ε*^2^).

Langmuir and Freundlich models were adopted for isothermal adsorption model [19].

(5) $$\begin{array}{c} \text{Langmuir model}=\frac{\rho_{i}}{q}=\frac{\rho_{i}}{q_{n}}+\frac{1}{q_{n}k_{l}}, \end{array}$$

(6) $$\begin{array}{c} \text{Freundlich model}=\mathrm{lg}q=\mathrm{lg}k_{e}+\frac{1}{n}\mathrm{lg}\rho_{i}. \end{array}$$

Langmuir model dimensionless separation factor is as follows:

(7) $$\begin{array}{c} R_{L}=\frac{1}{1+k_{l}\rho }, \end{array}$$

where *q* denotes adsorption capacity (mg g^−1^); *q*_*n*_ denotes the maximum adsorption capacity (mg g^−1^); *k*_l_, *k*_*e*_, and *n* are the adsorption constant; and *R*_*L*_ indicates the properties of an adsorption process [20].

## 3. Results and Discussion

### 3.1. SEM Analysis

**Figure 1:**
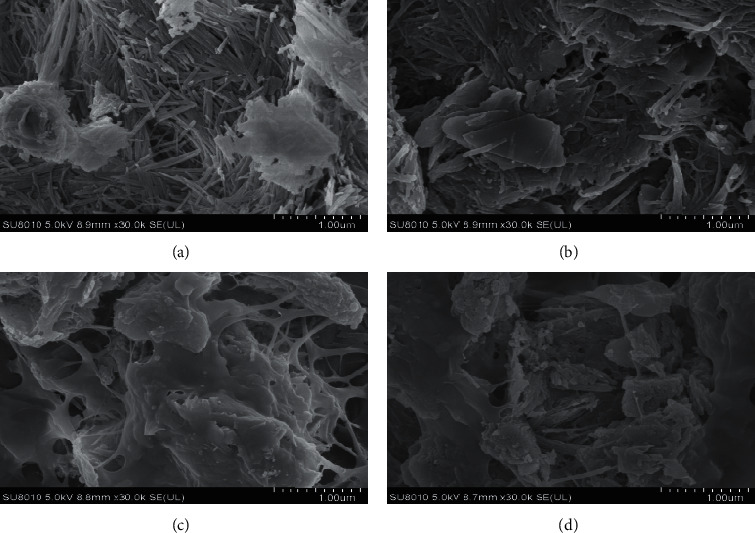
Sodium alginate colloid is 50,000 times before adsorption (a), 50,000 times after adsorption of As(III) (b), 50,000 times after adsorption of Pb(II) (c), and 50,000 times after adsorption of Zn(II) (d).

Figure 1 shows SEM images before and after the adsorption of As(III), Pb(II), and Zn(II) in waste water by sodium alginate gel. As can be seen, the morphology of sodium alginate gel before adsorption was mainly acicular and rod-like with large gaps between materials. After adsorption, the needle-like and rod-like structures were significantly reduced with smaller gaps between the materials. Herein, numerous spherical particles were filled after the adsorption of metal ions. These observations and analyses revealed that sodium alginate gel removes metal ions through pore filling and adsorption on its inner surface. Figure 1 also illustrates that the physical and chemical adsorption reactions took place on the materials.

**Figure 2:**
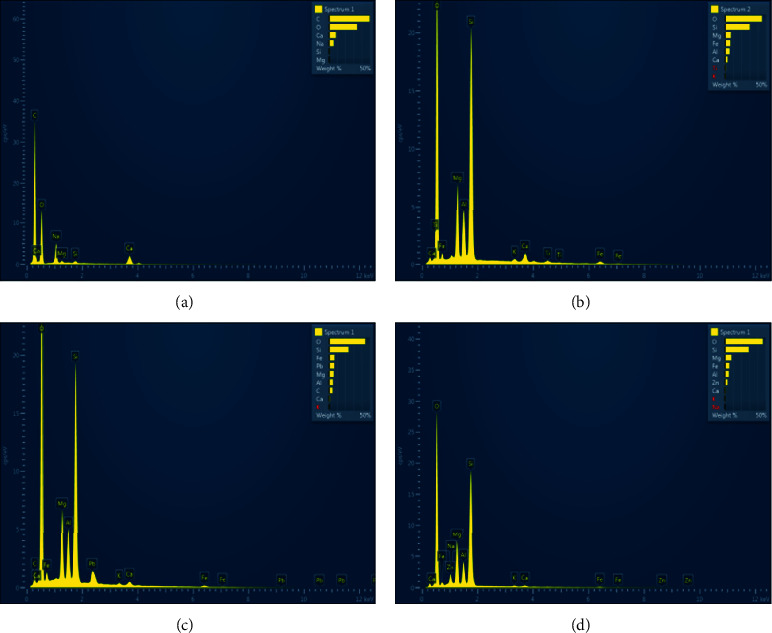
EDS photos of sodium alginate colloid before adsorption (a), after adsorption of As(III) (b), after adsorption of Pb(II) (c), and after adsorption of Zn(II) (d).

Figure 2 shows EDS photos before and after the adsorption of As(III), Pb(II), and Zn(II) by sodium alginate gel. Before adsorption, the alginate gel mainly composed of C, O, and Si elements. While, the adsorption peaks of Pb and Zn occurred after adsorption. Apparently, As adsorption was not as significant as those of Pb and Zn.

### 3.2. XPS Analysis

**Figure 3:**
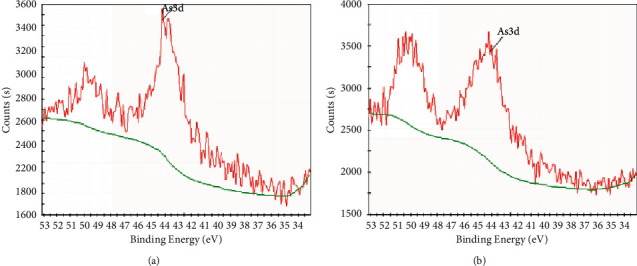
As(3d) XPS analysis results of sodium alginate before (a) and after (b) As adsorption.

Figure 3 shows As(3d) XPS high resolution spectra of sodium alginate gel before and after adsorption. After adsorption, there was no new peak of As(3d), and the area of energy loss peak increased. As(3d) exists as As(2S3) (at 44.18 eV, photoelectronic strength of 3960.68 Counts·s^−1^) and As(loss) (at 50.38 eV, Optoelectronic strength 3669.54 Counts·s^−1^). As can be seen from the existing peak area of As(3d), the content ratio of As(2S3)/As(loss) was about 1 : 1. It can be concluded that the adsorption reaction of As is dominated by physical adsorption; As is adsorbed on the surface of the gel; the redox site and the adsorption acid site of the gel were reduced that, in turn, inhibited the subsequent adsorption process of As and reduced the adsorption activity of the gel.

**Figure 4:**
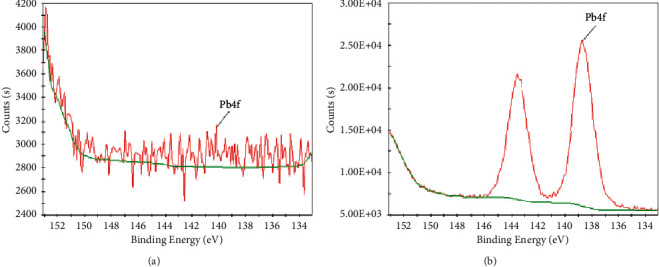
Pb(4f) XPS analysis results of sodium alginate before (a) and after (b) Pb adsorption.

Figure 4 shows the Pb(4f) XPS high-resolution spectra of sodium alginate gel before and after the adsorption of sodium alginate gel with lead nitrate solution. As can be seen, a new peak of Pb(4f) appeared after adsorption. Pb(4f) exists as Pb(4f5) (at 143.58 eV, photoelectron intensity 21617.6 Counts·s^−1^) and Pb(Ntv Ox) (at 138.68 eV, optoelectronic strength 25628.4 Counts·s^−1^). It also revealed the formation of intrinsic oxide of lead on the surface of the gel, suggesting that the adsorption is dominated by chemisorption and more stable.

**Figure 5:**
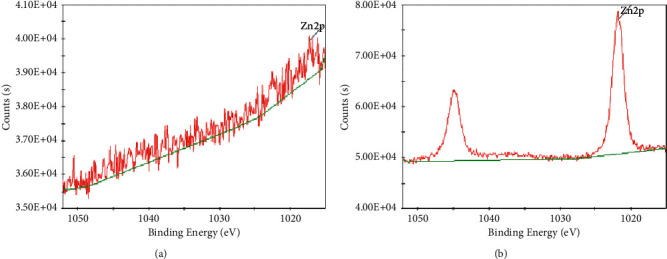
Zn(2p) XPS analysis results of sodium alginate before (a) and after (b) Zn adsorption.

Figure 5 shows the Zn(2p) XPS high-resolution spectra of sodium alginate gel before and after adsorption with zinc nitrate hexahydrate solution. A new peak of Zn(2p) occurred after adsorption. Zn(2p) exists as Zn(2p1) (at 1044.78 eV, optoelectronic strength 63308.3 Counts·s^−1^) and Zn(2p3) (at 1021.78 eV, optoelectronic Strength 78658.9 Counts·s^−1^). The peak area of Zn(2p) indicates that the content ratio of Zn(2p1)/Zn(2p3) was about 4 ∶ 3. This indicates the formation of hydroxyl complexes of Zn on the surface of the gel by chemisorption, which is more favorable for adsorption.

### 3.3. FTIR Analysis

**Figure 6:**
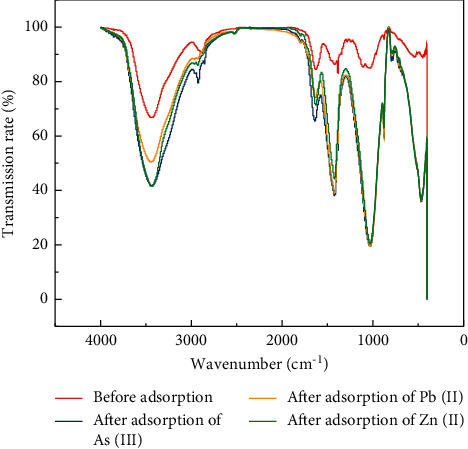
FTIR spectra of sodium alginate gel before adsorption, after adsorption of As(III), Pb(II), and Zn(II).

FTIR spectra before and after adsorption are shown in Figure 6 and Table 1. The stretching vibration peak of −OH hydrogen bond between molecules is 3440.29 cm^−1^; the stretching vibration peak of −CH was 2924.10 cm^−1^; the stretching vibration peak of C=O on aromatic group was 1617.18 cm^−1^; C−H bending vibration (1465–1340 cm^−1^) was located at 1420.82 cm^−1^; the bending vibration peak of C−O of alcohols and phenols was 1031.15 cm^−1^; C−H bending vibration absorption occurred at 876 cm^−1^. Figure 6 shows that sodium alginate gel can adsorb effectively As(III), Pb(II), and Zn(II), and that causes the peak to change. Altogether, these results indicate that the adsorption reaction is not a single physical or chemical reaction.

### 3.4. Kinetic Adsorption of As(III), Pb(II), and Zn(II) by Sodium Alginate Composite Gel

**Figure 7:**
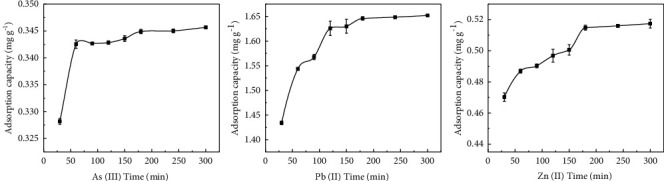
Influence of sodium alginate composite gel on the adsorption time of As(III), Pb(II), and Zn(II).

The effects of time on the adsorption of sodium alginate composite gel for As(III), Pb(II), and Zn(II) are shown in Figure 7. The adsorption time reflects both the cost and the efficiency adsorption process [21]. The adsorption capacity of As(III), Pb(II), and Zn(II) by sodium alginate composite gel increased with time. As shown in Figure 7, the adsorption of As(III) by sodium alginate composite gel increased rapidly between 0 and 50 min, and slight increase happened in the amount of adsorption after 150 min; the adsorption capacity of sodium alginate composite gel for Pb(II) increased rapidly between 0 and 120 min, but it increased slightly after 120 min; the adsorption capacity of Zn(II) increased rapidly from 0 to 200 min and followed by a fluctuation. Rapid adsorption capacity of the metals at early stage of adsorption process is due to the existence of vacant adsorption sites on the upper surface of the adsorbent along with the greater interaction between adsorbents. At the later stage of adsorption, the number of available adsorption sites on the adsorbent surface decreased, and the metal ions gradually spread inward until the saturation of active sites. Thus, equilibrium was reached and the adsorption rate decreased [22–25].

Adsorption kinetics model was used to fit the adsorption data of composite gels at different times, and the fitting data are summarized in Table 2. In accordance, the data of adsorption of As(III), Pb(II), and Zn(II) by sodium alginate composite gel were better fitted with the pseudo-second-order kinetic model (*R*^2^ > 0.9545) compared with the pseudo-first-order kinetic model (*R*^2^ < 0.9598). Our results suggested that the adsorption process of As(III), Pb(II), and Zn(II) by sodium alginate composite gel occurred mainly via chemisorption.

### 3.5. Isothermal Adsorption of As(III), Pb(II), and Zn(II) by Sodium Alginate Composite Gel

**Figure 8:**
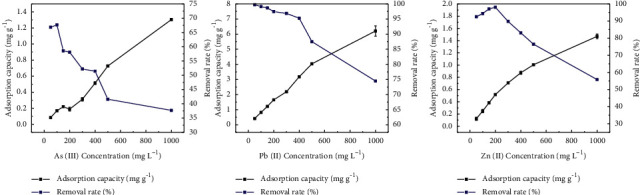
Isothermal adsorption of As(III), Pb(II), and Zn(II) by sodium alginate composite gel.

As shown in Figure 8, the removal rate of As(III) increased and decreased at its solution concentrations less than 100 mg L^−1^ and 100–500 mg L^−1^, respectively. The removal rate of As(III) decreased slowly at its solution concentrations above 500 mg L^−1^. The removal rate of Pb(II) decreased slowly and rapidly at its solution concentration less than 400 mg L^−1^ and 500–1000 mg L^−1^, respectively. The removal rate of Zn(II) increased slowly and decreased rapidly at its solution concentrations of less than 200 mg L^−1^ and between 200 and 1000 mg L^−1^, respectively. The adsorption capacity of As(III), Pb(II), and Zn(II) by sodium alginate composite gel to increased continuously with increasing their solution concentrations. With the increase of the initial concentration of metal ions, the equilibrium adsorption capacity increases at the same time. The reason is that the gradually increasing concentration of metal ions increases the chance of collision between ions and gel and enhances the adsorption effect [26]. The quality and number of active sites of sodium alginate composite gel were fixed with the increase of metal ion concentration; excessive metal ions tended to block the available active sites, thus preventing the subsequent ions to enter the adsorptive active sites. Therefore, the rate of adsorption capacity slowed down and the removal rate decreased [27–30]. This is consistent with the results of Masoumi et al. [31].

Langmuir and Freundlich models were used to fit the data of As(III), Pb(II), and Zn(II) (Table 3). In accordance with it, the Langmuir model could better describe the adsorption of Zn(II) by sodium alginate composite gel, and the Freundlich model could better describe the adsorption of As(III) and Pb(II). The maximum adsorption capacities of As(III), Pb(II), and Zn(II) by Langmuir isothermal adsorption model were 3.26, 11.723, and 1.607 mg g^−1^, respectively. Our results also indicated that sodium alginate composite gel removed Zn(II) via monolayer adsorption, As(III) and Pb(II) via multilayer adsorption. In Langmuir isothermal adsorption model, the *R*_*L*_ values of As(III), Pb(II), and Zn(II) by sodium alginate composite gel were less than 1, indicating that sodium alginate composite gel is favorable for the adsorption of As(III), Pb(II), and Zn(II).

### 3.6. Impact of Temperature on the Adsorption of As(III), Pb(II), and Zn(II) by Sodium Alginate Composite Gel

**Figure 9:**
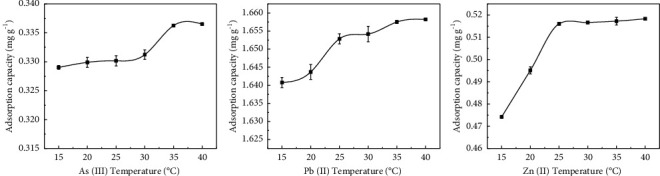
Adsorption effects of sodium alginate composite gel on As(III), Pb(II), and Zn(II) at different temperatures.

The adsorption effects of sodium alginate composite gel on As(III), Pb(II), and Zn(II) in relation to the different temperatures are shown in Figure 9. Apparently, temperature is an important parameter to adsorb metal ions, affecting solid-liquid interface, swelling property of adsorbent, and fluidity of metal ions [21]. The present study set the temperature to 20, 25, 30, 35, 40, and 45°C. We found that with the increasing the temperature the adsorption capacity of sodium alginate composite gel on metal ions increased gradually, indicating an endothermic reaction between the adsorbent and the metal ions. At temperatures above 25°C, the adsorption capacity of Pb(II) and Zn(II) increased slightly, while that of As(III) experienced slight changed at temperature above 35°C. A possible explanation is that an increase in temperature promotes the migration and diffusion of metal ions in the solution [32]. Correspondingly, this increases the kinetic energy and promotes the diffusion of metal ions to the adsorbent. Similar results were observed for the adsorption of metal ions by other adsorbent materials [33–35].

### 3.7. Impact of Adsorbent Dosage on the Adsorption of As(III), Pb(II), and Zn(II) by Sodium Alginate Composite Gel

**Figure 10:**
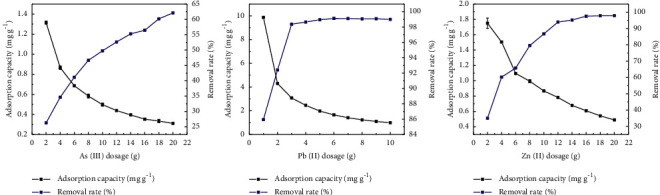
Adsorption effects of sodium alginate composite gel on As(III), Pb(II), and Zn(II) with different addition amounts.

The adsorption effects of different doses of sodium alginate composite gel on As(III), Pb(II), and Zn(II) are shown in Figure 10. Sodium alginate gel material was added to 50 mL of wastewater at a concentration of 200 mg L^−1^. Accordingly, the removal rate of As(III), Pb(II), and Zn(II) increased with increasing the amount of sodium alginate gel, the amount of adsorption decreased accordingly. The removal rate of wastewater As(III) tended to be flat at sodium alginate composite gel dosage of 20 g. At adsorbent mass of 4 g, negligible changes occurred in the removal rate of Pb(II). At the adsorbent mass between 2 g and 12 g, rapid increases occurred in the removal rate of wastewater Zn(II) followed by a slight increase. The slow removal efficiency observed at low sorbent dosage might be attributed to the presence of a limited number of active adsorbent sites [36]. However, the amount of adsorption reduced with increasing the mass of adsorbents. This might be justified by the fact all adsorbent sites were not occupied. Similar results were observed for the removal of metal ions via chitosan-tripolyphosphate beads [37].

### 3.8. Impact of Solution pH on the Adsorption of As(III), Pb(II), and Zn(II) by Sodium Alginate Composite Gel

**Figure 11:**
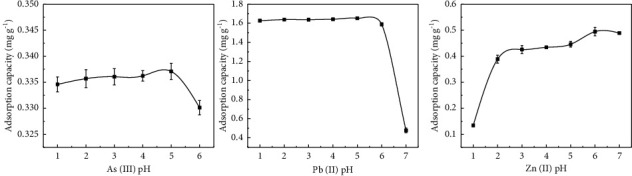
Adsorption effects of sodium alginate composite gel on As(III), Pb(II), and Zn(II) at different pH values.

Figure 11 shows the adsorption efficacy of solution sodium alginate composite gel for As(III), Pb(II), and Zn(II) at different pH values. The solution pH for As(III), Pb(II), and Zn(II) ranged from 1–6, 1–7, and 1–7, respectively. At solution pH > 7, flocculent material precipitate was generated in As(III), Pb(II), and Zn(II) solutions. Hence, this experiment set the maximum pH value at 7. At solution pH < 4, As(III) adsorption increased and reached its peak at solution pH of 5. At solution pH of 5–6, gradual decreases occurred in the adsorption rate. At solution pH > 6, rapid decreases were found in the adsorption rate, because in the form of H_3_AsO_3_, As(III) is electrically neutral. Hence, it was less affected by solution pH between 1 and 6 [38]. At solution pH 1–5, Pb(II) adsorption capacity increased slowly and reached its peak value at pH 5 and then decreased rapidly. This is due to the precipitation of lead ions at solution pH about 7. Rapid increase took place in Zn(II) adsorption capacity at solution pH 1–3, while a slight increase was observed in its adoption at pH of 3–6. The Zn(II) adsorption reached its peak at solution pH of 6, and it decreased together with the generation of flocculants. At low solution pH, the amount of metal adsorption amount was relatively small due to the high concentration of H^+^ that competes with metal ions for adsorption sites. In addition, H^+^ protonizes the surface of the gel, causing the repulsion of heavy metal ions [39]. At high solution pH, OH^−^ form hydrative hydroxyl complexes with metal ions, thus reducing the adsorption capacity [40].

### 3.9. Reusability of Sodium Alginate Composite Gel

**Figure 12:**
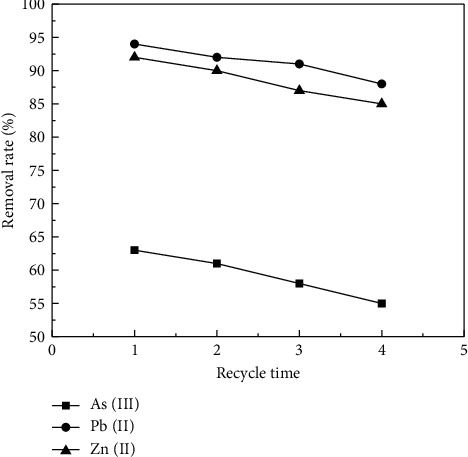
The removal efficiency of sodium alginate composite gel for wastewater As(III), Pb(II), and Zn(II) after four adsorption-desorption cycles.

To examine the reusability of sodium alginate gels, we conducted a desorption experiment using 0.05 mol·L^−1^ HNO_3_. As shown in Figure 12, the sodium alginate gel still had 55%, 88%, and 85% adsorption rate of As(III), Pb(II), and Zn(II) after four desorption experiments. This validates the strong reusability of sodium alginate colloids. The decrease in the removal rate may be due to the weight loss of the sodium alginate gel after four desorption experiments [41].

## 4. Conclusions

1. The surface functional groups were determined by SEM, EDS, XPS, and FTIR. The experimental results indicated that the adsorption of As(III), Pb(II), and Zn(II) by sodium alginate composite gel took place via both physical and chemical reactions.
2. The pseudo-second-order kinetic equation could better explain the adsorption effect of sodium alginate composite gel on As(III), Pb(II), and Zn(II). The Freundlich isothermal adsorption model could fit well the adsorption mechanism of Pb(II) and Zn(II), and the Langmuir model could better explain the adsorption mechanism of As(III), suggesting that the gel adsorption of As(III) was monolayer, and the adsorptions of Pb(II) and Zn(II) were multilayer.
3. The adsorption effect of sodium alginate composite gel on As(III), Pb(II), and Zn(II) gradually increased with increasing the temperature, oscillation time, and adsorbent dosage and finally tended to be stable. At solution pH of 6, sodium alginate composite gel had a good adsorption effect on As(III), Pb(II), and Zn(II).

## Figures and Tables

**Table 1:** Position of the main peaks in FTIR spectra of sodium alginate gel before adsorption, after adsorption of As(III), Pb(II), and Zn(II).

Wavenumber before sodium alginate gel adsorption (cm^−1^)	Wavenumber after sodium alginate gel adsorption (cm^−1^)			Assignment
	As(III)	Pb(II)	Zn(II)	
3440	3440	3443	3439	−OH stretching vibration
2924	2922	2922	2922	−CH stretching vibration
1617	1637	1638	1628	C=O stretching vibration
1420	1420	1420	1420	C−H bending vibration
1031	1027	1027	1027	C−O stretching vibration
876	876	877	877	C−H bending vibration

**Table 2:** Fitting parameters of three kinetic models for As(III), Pb(II), and Zn(II) adsorption by sodium alginate composite gel.

	Parameters	As(III)	Pb(II)	Zn(II)
Pseudo-first-order kinetic model	*q*_*e*_ (mg g^−1^)	0.3440	1.6225	0.4936
	*k*_1_ (min^−1^)	0.1025	0.0690	0.0008
	*ε* ^2^	0.0001	0.0013	0.0031
	*R* ^2^	0.9567	0.7707	0.9598

Pseudo-second-order kinetic model	*q*_*e*_ (mg g^−1^)	0.3514	1.6569	0.5252
	*k*_2_ (min^−1^)	0.0097	0.0156	0.0957
	*ε* ^2^	0.0001	0.0003	0.0001
	*R* ^2^	0.9874	0.9545	0.9841

**Table 3:** Fit parameters of three isothermal models for the adsorption of As(III), Pb(II), and Zn(II) by sodium alginate composite gel.

	Langmuir model				Freundlich model			
	*q*_*n*_ mg·g^−1^	*k*_l_ mg·l^−1^	*R* ^2^	SE	*k*_*e*_	1/*n*	*R* ^2^	SE
As(III)	3.260	0.001	0.580	15.963	100.786	0.742	0.861	0.460
Pb(II)	11.723	0.019	0.817	1.548	1.076	0.356	0.870	0.902
Zn(II)	1.607	0.026	0.884	10.865	6.079	0.379	0.735	1.025

## Data Availability

The data used to support the findings of this study are included within the article.
